# Non-target Site Herbicide Resistance Is Conferred by Two Distinct Mechanisms in Black-Grass (*Alopecurus myosuroides*)

**DOI:** 10.3389/fpls.2021.636652

**Published:** 2021-03-03

**Authors:** Sara Franco-Ortega, Alina Goldberg-Cavalleri, Andrew Walker, Melissa Brazier-Hicks, Nawaporn Onkokesung, Robert Edwards

**Affiliations:** Agriculture, School of Natural, and Environmental Science, Newcastle University, Newcastle upon Tyne, United Kingdom

**Keywords:** herbicide metabolism, pendimethalin, fenoxaprop, xenome, black-grass, weighted gene co-expression network analysis (WGCNA), non-target site resistance (NTSR)

## Abstract

Non-target site resistance (NTSR) to herbicides in black-grass (*Alopecurus myosuroides*) results in enhanced tolerance to multiple chemistries and is widespread in Northern Europe. To help define the underpinning mechanisms of resistance, global transcriptome and biochemical analysis have been used to phenotype three NTSR black-grass populations. These comprised NTSR1 black-grass from the classic Peldon field population, which shows broad-ranging resistance to post-emergence herbicides; NTSR2 derived from herbicide-sensitive (HS) plants repeatedly selected for tolerance to pendimethalin; and NTSR3 selected from HS plants for resistance to fenoxaprop-*P*-ethyl. NTSR in weeds is commonly associated with enhanced herbicide metabolism catalyzed by glutathione transferases (GSTs) and cytochromes P450 (CYPs). As such, the NTSR populations were assessed for their ability to detoxify chlorotoluron, which is detoxified by CYPs and fenoxaprop-P-ethyl, which is acted on by GSTs. As compared with HS plants, enhanced metabolism toward both herbicides was determined in the NTSR1 and NTSR2 populations. In contrast, the NTSR3 plants showed no increased detoxification capacity, demonstrating that resistance in this population was not due to enhanced metabolism. All resistant populations showed increased levels of *Am*GSTF1, a protein functionally linked to NTSR and enhanced herbicide metabolism. Enhanced *Am*GSTF1 was associated with increased levels of the associated transcripts in the NTSR1 and NTSR2 plants, but not in NTSR3, suggestive of both pre- and post-transcriptional regulation. The related HS, NTSR2, and NTSR3 plants were subject to global transcriptome sequencing and weighted gene co-expression network analysis to identify modules of genes with coupled regulatory functions. In the NTSR2 plants, modules linked to detoxification were identified, with many similarities to the transcriptome of NTSR1 black-grass. Critical detoxification genes included members of the CYP81A family and tau and phi class GSTs. The NTSR2 transcriptome also showed network similarities to other (a)biotic stresses of plants and multidrug resistance in humans. In contrast, completely different gene networks were activated in the NTSR3 plants, showing similarity to the responses to cold, osmotic shock and fungal infection determined in cereals. Our results demonstrate that NTSR in black-grass can arise from at least two distinct mechanisms, each involving complex changes in gene regulatory networks.

## Introduction

Black-grass (*Alopecurus myosuroides*) is an annual grass weed of cereals that is widely dispersed in genetically diverse populations across Western Europe (Moss, 1979). Herbicide resistance is now widespread in these populations; and in the United Kingdom, the respective loss of weed control incurs an economic cost of ∼0.5 bn GBP/year, being associated with 1 million ton/year of yield loss in wheat production (Varah et al., 2020).

Within these resistant populations, non-target site resistance (NTSR) is commonly encountered and particularly difficult to combat, as it contributes to loss of control of many pre- and post-emergence selective herbicides, irrespective of their chemistry or mode of action (Preston, 2004). NTSR is a complex, multigenic trait that invokes diverse resistance mechanisms to herbicides and is linked to their reduced uptake, translocation, and enhanced detoxification, as well as less-well-understood broad-ranging cytoprotective mechanism (Gaines et al., 2010; Délye, 2013). Elevated herbicide detoxification, which is also known as enhanced metabolic resistance (EMR), is one of the best studied mechanisms of NTSR in wild grasses, including rigid ryegrass (*Lolium rigidum*), wild oat (*Avena fatua*), and black-grass (Délye et al., 2011; Délye, 2013). Central to EMR is the enhanced expression of proteins involved in herbicide detoxification, which includes cytochromes P450 (CYPs), glutathione transferases (GSTs), UDP-glycosyltransferases (UGTs), and ATP-binding cassette transporters (ABC transporters). These proteins act collectively to detoxify herbicides (Délye, 2013). In contrast to EMR, the molecular components of other NTSR mechanisms remain largely unknown.

In a previous study, using quantitative proteomics (Tétard-Jones et al., 2018), we identified three “types” of NTSR, namely, (1) multiple resistance to herbicides with differing modes of action (NTSR1), (2) cross-resistance to chemistries acting on the same mode of action (NTSR2), and (3) resistance to a specific herbicide chemistry (NTSR3). The classification of these overall NTSR types was made possible by characterizing populations that had been selected for through repeated selections with specific herbicides. The NTSR1 plants were derived from the field-derived Peldon population that had evolved resistance over generations of exposure to multiple herbicide classes. The other types of NTSR black-grass plants were derived from the herbicide-sensitive (HS) Rothamsted population, a lineage of black-grass that has never been exposed to herbicides. The NTSR2 plants were generated by a repeated selection with the pre-emergence herbicide pendimethalin, whereas the consecutive selection of the same HS plants for resistance using the post-emergence herbicide fenoxaprop-*P*-ethyl yielded a NTSR3 population. Previous studies have shown that elevated levels of the phi (F) class GST *Am*GSTF1 were integrally linked to NTSR in black-grass (Cummins et al., 2013), with proteomics showing enhanced levels of the protein in resistant populations (Tétard-Jones et al., 2018). Proteomics also demonstrated that additional proteins were induced in the NTSR1 and NTSR2 plants that differed from those in the NTSR3 population (Tétard-Jones et al., 2018). Cumulatively, these results indicate the presence of unknown NTSR mechanisms in these black-grass populations.

**FIGURE 1:**
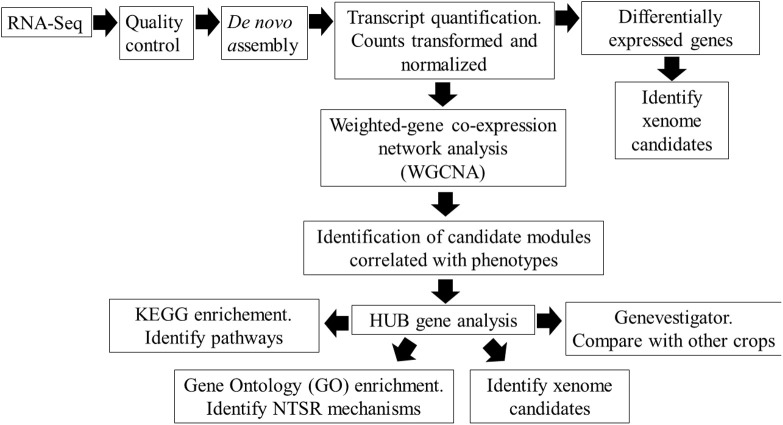
Overview of the workflow for identifying the non-target site resistance (NTSR) mechanisms in black-grass populations using RNA-Seq analysis.

To explore these NTSR mechanisms in greater detail and to reduce the effects of background genetic diversity in differing black-grass populations, we have compared the global transcript expression profiles of the NTSR2 and NTSR3 plants with those in the parent HS populations using a tiered approach (Figure 1). In particular, we have applied a weighted gene co-expression network analysis (WGCNA), a powerful tool to study regulatory transcriptional networks within transcriptome datasets, which also helps identify key genes that underpin core mechanisms (Zhang and Horvath, 2005; Horvath, 2011). In the current study, WGCNA subdivides interconnected genes into modules that can be correlated with the differing types of NTSR. Core to our analysis, we have performed WGCNA on the related HS, NTSR2, and NTSR3 plants and compared the differentially regulated genes to those present in the field-derived Peldon NTSR1 population, allowing us to compare the transcriptomes of all three NTSR subtypes. In each case, we have determined changes in latent gene expression in each population of resistant black-grass and have not taken into account the active response of the plants to herbicide exposure. While the induction and suppression of specific genes in response to chemical stress would have been insightful, the differences in resistance profile to the multiple herbicides used in the study rendered this approach impractical.

## Materials and Methods

### Black-Grass Populations and Plant Growth Conditions

The NTSR2 and NTSR3 plants were generated from the HS population by selecting for survivors from repeated field-rate applications of pendimethalin (×8) and fenoxaprop-*P*-ethyl (×6), respectively, over consecutive growing seasons. The resulting NTSR2 and NTSR3 plants were assessed for their tolerance to herbicides acting on acetyl-CoA carboxylase (ACCase), acetolactate synthase (ALS), tubulin assembly, and fatty acid elongation, respectively (Marshall et al., 2013). The NTSR3 plants were only resistant toward the ACCase herbicide fenoxaprop-*P*-ethyl, while the NTSR2 population was resistant toward pendimethalin (inhibitor of tubulin assembly) and ACCase inhibitors (including fenoxaprop-*P*-ethyl), but not to compounds acting on ALS (Tétard-Jones et al., 2018). Neither of the resistant populations showed evidence of TSR-linked mutations in the respective ACCase or ALS genes (Marshall et al., 2013). The field-derived Peldon plants were used as a well-characterized reference NTSR1 black-grass population demonstrating EMR and showing cross-resistance to ACCase, tubulin assembly, and photosystem II inhibitors including fenoxaprop-*P*-ethyl, diclofop-methyl fenoxaprop-*P*-ethyl, fluazifop-*P*-butyl, tralkoxydim, cycloxydim, pendimethalin, and chlorotoluron (Hall et al., 1997; Marshall et al., 2013). Peldon plants are also reported to show insensitivity to ALS inhibitors such as iodosulfuron and mesosulfuron due to mutations in the targeted ALS gene (Marshall and Moss, 2008).

Black-grass seeds were pre-germinated in petri dishes on four layers of filter paper (Whatman No. 1, Sigma Aldrich, Gillingham, United Kingdom) wetted with 7 ml of sterile deionized water. Petri dishes were maintained at 4°C in the dark for 7 days prior to transfer to a growth cabinet with a 16 h light/8 h dark with intensity of 220 μmol m^–2^ s^–1^ and 18°C/16°C temperature cycle. For each population, five seedlings were sown into 10 cm plastic pots (*n* = 5) containing John Innes No. 2 compost (East Riding Horticulture, York, United Kingdom), mixed with Osmocote slow release fertilizer (Osmocote, Suffolk, United Kingdom), and propagated in a growth cabinet (Sanyo, MLR-351 SANYO Electric Co., Ltd., Osaka, Japan).

### Herbicide Metabolism Assays

Metabolism studies in black-grass were conducted using herbicides undergoing primary detoxification by either CYPs (the phenylurea chlorotoluron) or GSTs (the aryloxyphenoxypropionate fenoxaprop acid). Leaves from the HS, NTSR1, NTSR2, and NTSR3 plants, each at the two-tiller stage (4 weeks), were cut into 1 cm-long pieces and 150 mg of leaf material submerged in 25 ml of H_2_O containing 0.1% biopower (Bayer Crop Science, Leverkusen, Germany) and either 50 μM of chlorotoluron (Sigma Aldrich) or 50 μM of fenoxaprop acid (Sigma Aldrich). After 24 h, leaf material (*n* = 5) was collected, dried, and then frozen in liquid nitrogen prior to storage at -80°C. Samples were ground in liquid nitrogen and extracted with 80% methanol (750 μl, Sigma Aldrich) overnight at 4°C, prior to centrifugation (4,500×*g*, 4°C, 5 min). The supernatant (5 μl) was analyzed on a Waters Xevo G2-XS QTof mass spectrometer following electrospray ionization (Waters Ltd., Wilmslow, United Kingdom) as described (Davies et al., 2020). The chlorotoluron and fenoxaprop acid metabolites were identified from their reference spectra, with metabolites quantified based on calibration curves prepared from the respective parent herbicides [chlorotoluron parent [M–H]^+^ 213.0795, hydroxylated metabolite [M–H]^+^ 229.0744, and fenoxaprop acid form [M–H]^+^ 334.0482 and the respective glutathione-conjugated metabolite *S*−(6−chlorobenzoxazole−2−yl)−glutathione [M–H]^+^ 459.0741].

### Enzyme-Linked Immunosorbent Assay for AmGSTF1

Total protein from 3–5 five-leaf stage of untreated black-grass was extracted from 100 mg of leaf tissue as described by Comont et al. (2020). Total protein concentration was determined (Bio-Rad protein assay kit, Bio-Rad, Hercules, CA, United States) using bovine serum albumin (BSA; Sigma Aldrich) as a standard.

*Am*GSTF1 levels in protein samples were quantified by ELISA using specific sheep antibodies raised against *Am*GSTF1 in 96-well plates (Comont et al., 2020). Briefly, 96-well plates were coated with the primary antiserum (S909-D, diluted to 1 μg/ml in phosphate-buffered saline (PBS) for 16–18 h at 4°C. Plates were washed four times with wash buffer [PBS with 0.1% (v/v) Tween 20 (Sigma Aldrich)] before black-grass protein (100 μg) extracts were applied. After 1 h incubation, plates were washed four times, and the secondary antiserum conjugated with horseradish peroxidase (S908D-HRP diluted to 25 ng/ml) was applied and incubated for 1 h. Then, plates were washed four times, and afterward, colorimetric tetramethylbenzidine reagent (TMB; Sigma Aldrich) was applied. Reactions were terminated after 30 min with 3 M of HCl, and the absorbance at 450 nm was determined. *Am*GSTF1 protein concentration was calculated from a standard curve prepared from pure recombinant *Am*GSTF1 protein. Data were analyzed by one-way ANOVA, followed by Tukey’s honestly significant difference (HSD) *post hoc* test (SPSS version 26 software, IBM, Chicago, IL, United States). The assumption of homogeneity of variance for one-way ANOVA was tested by Levene’s test.

### RNA Extraction and Illumina Sequencing

The aerial tissue from untreated one to two tiller black-grass plants (4 weeks) were used for RNA extraction, analyzing five biological replicates of the HS, NTSR2, and NTSR3 populations, each comprising five co-harvested plants. Total RNA was extracted from frozen tissues, using NucleoSpin RNA plant kits (Macherey-Nagel, Düren, Germany) according to the manufacturer’s instructions. Library construction, Illumina sequencing, and *de novo* transcriptome assembly were performed by Genomics Services, Earlham Institute (Norwich, United Kingdom) as described in Supplementary Material. All the sequencing data were deposited in the Gene Expression Omnibus (GEO) of the National Center for Biotechnology Information (NCBI) (GSE162422).

### RNA-Seq *de novo* Assembly and Gene Functional Annotation

*De novo* transcriptome assembly was carried out with Trinity v2.8.5 using default parameters (Haas et al., 2013), with the trimmomatic option added to perform quality testing and adapter trimming (MacManes, 2014). Coding regions of assembled transcripts were identified using TransDecoder v5.5.0 (Haas et al., 2013) with default parameters and the “single_best_only” option to define a single open reading frame (ORF) per transcript.

High-throughput protein function annotations for all protein sequences were obtained using the human readable description (HRD) and Gene Ontology (GO) terms, with InterPro classification and predictive domain analysis. Proteins were annotated using the AHRD (Automated Assignment of Human Readable Descriptions v.3.3.3^1^) using settings to define HRDs in favor of GO predictions. BLASTp analyses were performed against the *Arabidopsis thaliana* TAIR10 reference protein dataset^2^ and the Viridiplantae protein sequences from UniProt, Swiss-Prot, and TrEMBL datasets (data downloaded on 30 January 2020). GoA mapping was obtained from UniProt^3^, and InterPro annotations were retrieved from the InterPro database^4^. AHRD annotation quality was measured through three characters, namely, (1) bit score of the blast results higher than 50% with an *e*-value lower than e-10; (2) represented and overlap of the blast results higher than 60%; and (3) top token score of the assigned HRD higher than 0.5.

### Analysis of Transcript Expression and Identification of Differentially Expressed Genes

The analysis of transcript expression level was performed using salmon with autodetect strandedness and validation mapping parameters. Tximport was used to transform the salmon expression count data into the DESeq dataset (Soneson et al., 2016) utilizing DESeq2 (version 1.26.0) (Love et al., 2014). The comparison was set using the HS black-grass population as the baseline/control condition. *P*-values of each comparison were then adjusted following a Benjamini–Hochberg analysis that controls the false discovery rate (FDR) (Storey and Tibshirani, 2003). Genes counted as *n* < 1 across all the samples were removed. Counts were transformed and normalized using the DESeq2 package version. Principal component analysis (PCA) was performed with all the normalized counts using the function prcomp with the center and scale options of the package stats and plotted using the fviz pca ind function of the factoextra package version in R environment.

### Analysis of Herbicide Detoxification and Putative Non-target Site Resistance Biomarker Genes

Pairwise comparison of differences in the transcript expression of NTSR2 vs. HS and NTSR3 vs. HS was analyzed. Upregulated and downregulated genes were identified using the cut-off of log_2_FC ≥ 1 and adjusted *P*-value (FDR) ≤ 0.05, and log_2_FC ≤ 1 and adjusted *P*-value (FDR) ≤ 0.05, respectively. Detoxification differentially expressed genes (DEGs) including CYPs, GSTs, UGTs, and ABC transporters were identified for each comparison. ORFs of all detoxification (xenome) proteins were compared with those in the NCBI^5^ database using BLASTp^6^ in order to identify the xenome families.

The upregulation in xenome genes, notably members of the CYP and GST families in NTSR2 and NTSR3, were compared with that reported in the NTSR1 Peldon population derived from ion torrent next-generation sequencing (Tétard-Jones et al., 2018). In addition, the protein sequence of the different isoforms of the *Am*GTSF1 (phi family) identified by Cummins et al. (2013) were searched and identified by BLASTp within the GSTFs differentially expressed in each population. Due to the diversity of CYPs, a phylogenetic analysis was conducted to classify family and subfamily members in black-grass, as described in Supplementary Material. The tau (U) GSTs identified were compared with the respective genes found to be enhanced in Peldon NTSR1 plants including *Am*GSTU1, *Am*GSTU2a, *Am*GSTU2b, *Am*GSTU3, *Am*GSTU4, *Am*GSTU5, *Am*GSTU6, and *Am*GSTU7 (Nandula et al., 2019). In addition to the detoxification genes, putative NTSR biomarkers associated with the previous proteomic analyses of these populations were also analyzed (Tétard-Jones et al., 2018). These included the stem-specific protein TSJT1, 12-oxophytodienoate reductase 1 (OPR-1), D-3-phosphoglycerate dehydrogenase 1, NAD-dependent epimerase/dehydratase, and NADPH quinone oxidoreductase.

### Weighted Gene Co-expression Network Analysis

Co-expression networks were constructed using the WGCNA package (version 1.69) (Langfelder and Horvath, 2008). Normalized counts were filtered in function of variance (cut-off 0.8), keeping 54,728 unigenes where abundance was then log-transformed before performing the WGCNA analysis^7^. To satisfy the approximate scale-free network distribution criterion (Zhang and Horvath, 2005), the soft threshold power β was used to raise the co-expression similarity matrix and assess adjacency from 1 to 20 (Langfelder and Horvath, 2008) using the PickSoftThreshold function. A value of 18 was chosen to fulfill the scale-free topology criterion^8^. Afterward, the topological overlap measure (TOM) and the correspondent dissimilarity matrix (1—TOM) were calculated using the bicor correlation, which is based on an adjacency matrix, and it has been demonstrated to be more powerful and robust than Spearman correlation and Pearson pairwise analysis (Song et al., 2012). For each module of coregulated genes, a specific color was assigned to visualize the results.

### Identification of Non-target Site Resistance Modules

To identify modules associated with the respective NTSR phenotypes, the eigengene modules were calculated and correlated with the herbicide resistance trait of each population using a Pearson correlation to generate a heatmap. Modules correlating with a *P*-value < 0.05 were selected for further characterization.

### Identification of HUB Genes and Functional Annotation

To identify transcripts linked to specific NTSR traits, the respective “HUB” genes were identified within the selected modules, as unigenes having an absolute value of module membership (MM) >0.8 and a gene significance (GS), Pearson correlated with the phenotypic traits of >0.2. Common annotations of HUB unigenes between modules were then retrieved to identify potential common mechanisms linked to specific NTSR phenotypes. Venn diagrams representing the annotations of the HUB genes were then plotted using the function Venn diagram of the Venn Diagram package in R environment. To further characterize HUB genes, GO, and Kyoto Encyclopedia of Genes and Genomes (KEGG) enrichment of the respective gene list from each selected module was performed using topGO package in R (version 2.38.1.) and ClusterProfiler package (version 3.14.3), respectively. In each case, GO enrichment was performed using Fisher’s exact test with a node size of 5 and the weight01 algorithm (a mixture between the elim and weight algorithms). Genes associated with each enriched GO term were also determined. Only GO terms with *P*-value < 0.05 were further analyzed.

In order to perform the KEGG enrichment, the 199,761 protein sequences that formed the universe were used as query in the KAAS-KEGG Automatic Annotation Server (single-directional best hit method with the GHOSTX search) in order to assign a KO number. As the server limited the number of organisms selected as gene datasets, only 17 were selected (ath, cho, osa, dosa, ats, zma, psom, soe, bvg, lsv, han, oeu, nta, sly, vvi, gmx, and tcc), which represented 1,448,747 sequences. Pathways (ko) assigned to each KO were retrieved with the bitr_kegg function. Last, the enricher function was used for each module. Only pathways with *P*-value < 0.01 were analyzed.

Due to their known association with NTSR in black-grass, xenome genes (CYPs, GSTs, UGTs, and ABCs) and the eight molecular markers identified by Tétard-Jones et al. (2018) were also searched within the HUB genes for each key module.

### Quantitative Real-Time PCR

One microgram of RNA was used for cDNA synthesis using an iScript cDNA synthesis kit for RT-qPCR (Bio-Rad, United Kingdom) in a 20 μl reaction volume. Quantitative real-time PCR was performed using a Light Cycler 96 system (Roche, United Kingdom) in a total volume of 20 μl containing 1.5 μl of cDNA prepared from 1 μg of RNA, 10 μl of Luna universal qPCR master mix (New England Biolabs, United Kingdom), and 1.2 μl of 5 μM forward and reverse gene-specific primers. The reactions were run in a three-step program including melting curve analysis and initial incubation at 95°C for 10 min, followed by amplification for 40 cycles (95°C for 10 s, 59°C for 20 s, and 72°C for 30 s) and melting curve analysis from 72 to 95°C. The specific primers of ubiquitin from black-grass (*Am*UBQ; GenBank accession number: JN599096) were used for normalization. Relative transcript expression of *Am*GSTF1 (GenBank accession number: AJ010453) was calculated based on an efficiency corrected model (Pfaffl, 2001). Mean relative transcript expression from five biological replicates (*n* = 5) was used for statistical analysis (one-way ANOVA followed by Tukey’s HSD *post hoc* test, SPSS software version 26.0). Primer sequences are listed in Supplementary Table S1.

### Genevestigator Analysis

To investigate the potential links between NTSR mechanisms and changes in gene expression associated with other plant stress responses, the sequences of unigenes from HUB gene list identified in selected modules were blasted using local BLASTp (Camacho et al., 2009) against the genome of three cereals wheat (*Triticum aestivum* IWGSC), barley (*Hordeum vulgare* IBSC_v2), and maize (*Zea mays* B73_RefGen_v4) retrieved from ftp://ftp.ensemblgenomes.org/pub/plants/release-48/fasta/). Only the BLASTp results with *E*-values ≤ 0.01 were used for analysis. The Genevestigator (Hruz et al., 2008) signature tool of the orthologous genes identified by BLASTp in each of the crops was then used to interrogate for similarities in gene expression changes in 2,078 experiments in wheat, 912 in barley, and 2,536 in maize mRNASeq datasets. As the Genevestigator signature tool allowed a comparison using a query of 400 genes, only the genes with higher GS were included for each module.

## Results

### Differential Levels of Enhanced Herbicide Metabolism and AmGSTF1 in the Non-target Site Resistance Populations

**FIGURE 2:**
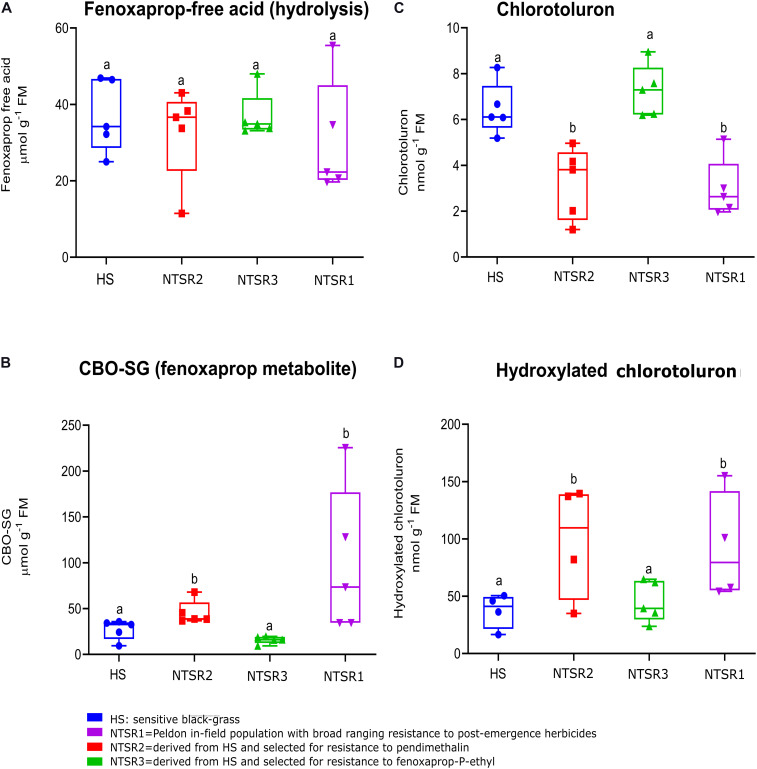
Enhanced detoxification of fenoxaprop-ethyl and chlorotoluron in non-target site resistance (NTSR) and herbicide-sensitive (HS) populations used in this study. The average level (*n* = 5) of **(A)** fenoxaprop free acid and **(C)** chlorotoluron in leaf tissues of black-grass populations at 24 h after herbicide treatment. An average level (*n* = 5) of **(B)** fenoxaprop metabolite and **(D)** chlorotoluron metabolite at 24 h after treatment. The levels of herbicides and the main metabolites were compared among HS, NTSR1, NTSR2, and NTSR3 populations by one-way ANOVA followed by Tukey’s honestly significant difference (HSD) *post hoc* test. Different letters indicate significant differences among populations (*P* ≤ 0.05).

To test for enhanced herbicide metabolism in the resistant black-grass plants, metabolism studies with the herbicides chlorotoluron and fenoxaprop acid were performed with all three NTSR and the HS populations over 24 h. These herbicides were selected as they undergo different routes of metabolism, with chlorotoluron being detoxified by ring hydroxylation catalyzed by CYPs, whereas fenoxaprop acid is acted on by GSTs, to form the *S*−(6−chlorobenzoxazole−2−yl)−glutathione (CBO-SG) conjugate. For each set of plants, the levels of the two parent herbicides and their respective primary detoxification products were determined in the leaves using a high-performance liquid chromatography (HPLC)–MS method (Davies et al., 2020). With chlorotoluron, as compared with the HS population, levels of parent herbicide were depleted in both the NTSR1 and NTSR2 plants, and this was accompanied by an increased accumulation of hydroxylated chlorotoluron (Figures 2C,D). In the case of fenoxaprop, while more of the CBO-SG glutathione conjugate was determined in NTSR1 and NTSR2, the levels of parent fenoxaprop were equivalent to those determined in the HS plants, suggesting the herbicide was in some type of equilibrium between soluble and insoluble forms (Figures 2A,B). In both cases, the significance of enhanced metabolite formation was confirmed by one-way ANOVA; *P*_(CBO__–__*SG)*_ = 0.02, *P*_(hydroxylated chloro__*to*__*luron)*_ = 0.04). In contrast, the NTSR3 plants showed no difference in metabolite formation with either herbicide as compared with the HS population (Figures 2B,D; one-way ANOVA; *P*_(CBO__–__*SG)*_ = 0.51, *P*_(hydroxylated chloro__*to*__*luron)*_ = 0.65). These results indicated that like the classic NTSR1 population, the NTSR2 plants exhibited EMR, while NTSR3 did not.

**FIGURE 3:**
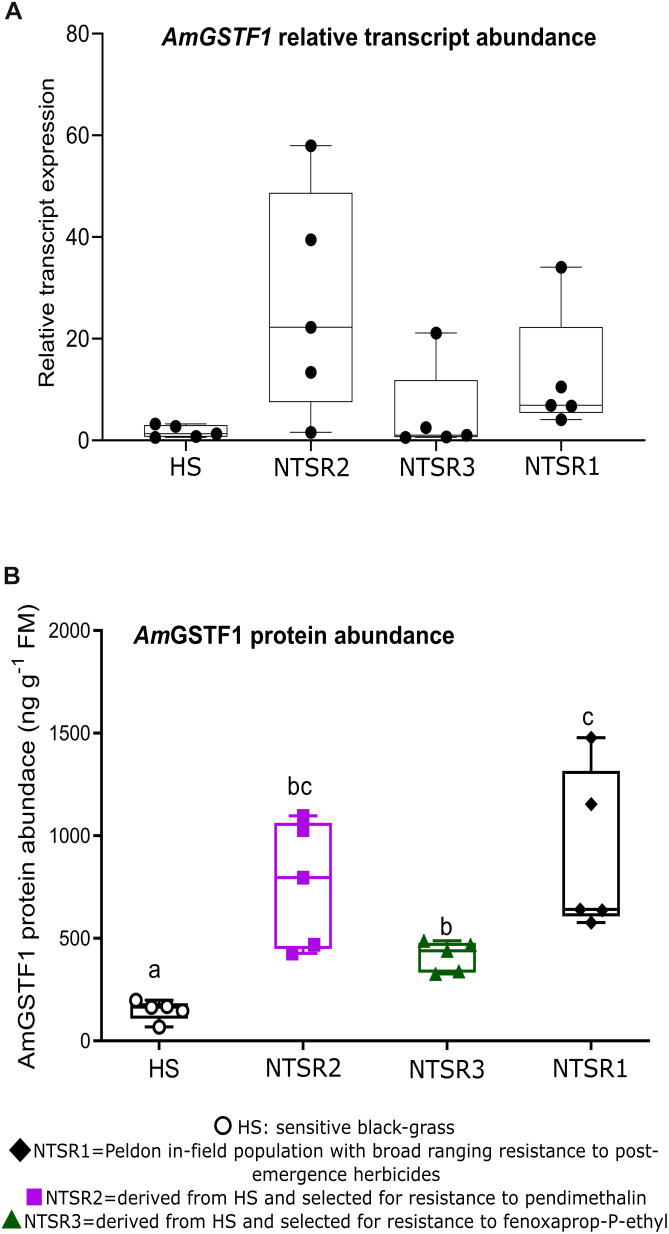
Multiple herbicide-resistant (MHR) black-grass populations accumulate constitutively high A*m*GSTF1 protein. **(A)** Average relative transcript expression (*n* = 5) of *Am*gstf1 and **(B)** *Am*GSTF1 protein abundance (*n* = 5) in herbicide-sensitive (HS), NTSR1, NTSR2, and NTSR3 populations. The relative expression and protein levels were compared among population by one-way ANOVA followed by Tukey’s honestly significant difference (HSD) *post hoc* test. Different letters indicate significant differences among populations (*P* ≤ 0.05).

As the enhanced accumulation of the biomarker *Am*GSTF1 is functionally and quantitatively linked to NTSR in black-grass populations (Cummins et al., 2013; Comont et al., 2020), the levels of this protein were determined by ELISA in each population, along with the quantitation by qPCR of the respective mRNA transcripts. As compared with the HS controls, the NTSR2 plants showed an elevated abundance of *Am*gstf1 transcripts, equivalent to that determined in the NTSR1 population (Figure 3A). In contrast, the *Am*gstf1 transcript expression in the NTSR3 population was not significantly different from that determined in HS plants (one-way ANOVA; *P* = 0.12). At the level of protein expression, all the NTSR populations contained higher concentrations of *Am*GSTF1 than the HS controls, with abundance being the greatest in the NTSR1 and NTSR2 plants (Figure 3B). The enhancement in *Am*GSTF1 protein, without a corresponding elevation in the respective transcripts in the NTSR3 plants, suggested that factors other than relative mRNA abundance must determine the content of this NTSR-diagnostic polypeptide in black-grass.

### Illumina Sequencing and *de novo* Assembly

Having confirmed that the NTSR2 and NTSR3 populations were displaying different resistance mechanisms, the respective plants were subjected to global RNA sequencing as referenced against their parental HS plants. *De novo* assembly resulted in the identification of a total of 299,782 unigenes, originating 579,893 transcripts (Supplementary Table S2). The N50, which corresponds with the length of the smallest contig in the dataset necessary to represent at least 50% of the assembly (Miller et al., 2010), was 943 and 1,567 bp for unigenes and transcripts, respectively, with a guanine–cytosine (GC) content of 48.63%. In total, 199,761 coding sequences were identified from the longest read from each identified ORF, with the reads quantified using Salmon (Supplementary Table S3) and annotated with respect to human readable (AHRD) and GO terms.

**FIGURE 4:**
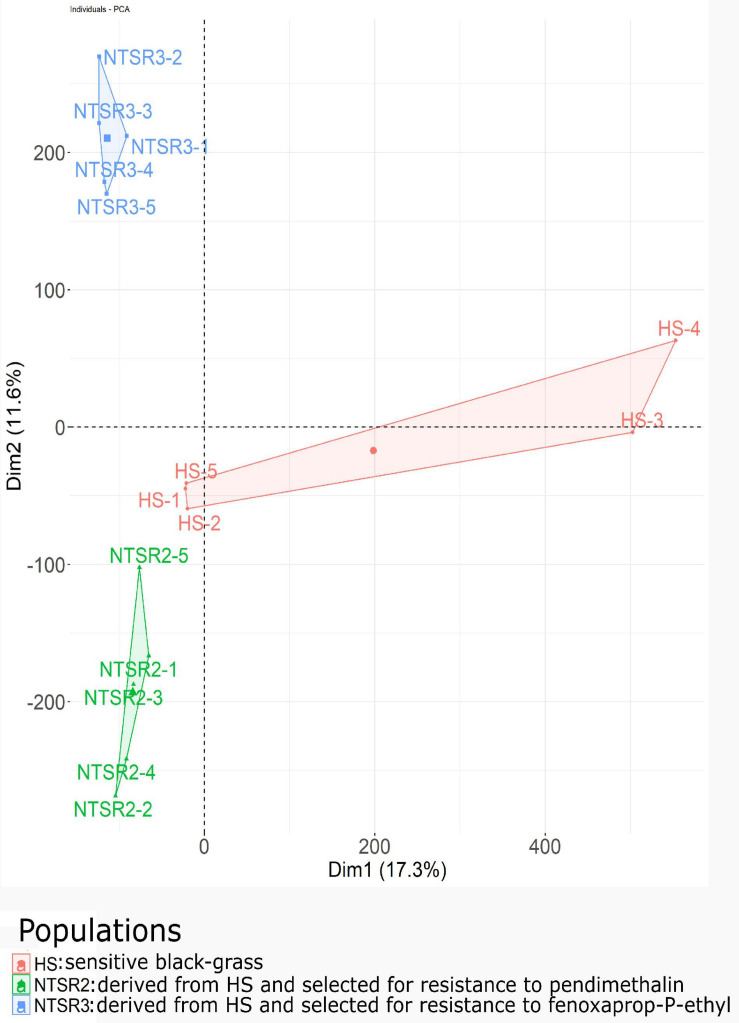
Principal component analysis of the global transcriptional expression profiles of the NTSR2 and NTSR3 black-grass populations were distinct from each other and from HS (Rothamsted) black-grass population. Each point represents one of the five biological replicates (each containing five plants) of each population. Axes represent the percentage of variance explained by each dimension, with the dimension 1 (Dim1) explaining 16.8% and the dimension 2 (Dim2) explaining 11.6% of the variance. HS, sensitive population; NTSR2, derived from HS and selected for resistance to pendimethalin; NTSR3, derived from HS and selected for resistance to fenoxaprop-*P*-ethyl.

The normalized unigenes were used to build a PCA after standardizing as a function of the NTSR2, NTSR3, and HS populations (Figure 4). While the PCA showed clear differences, the NTSR2 and HS populations showed several close associations. In contrast, the overall transcript expression of NTSR3 was clearly separated from that of the other two populations (principal component 2, 11.6% of the variation). The PCA further confirmed that the two NTSR populations had developed distinct transcriptomes that could not be explained by the natural genetic diversity of the populations.

### Differential Expression Pattern of Xenome Genes Observed in NTSR2 and NTSR3 Populations

**FIGURE 5:**
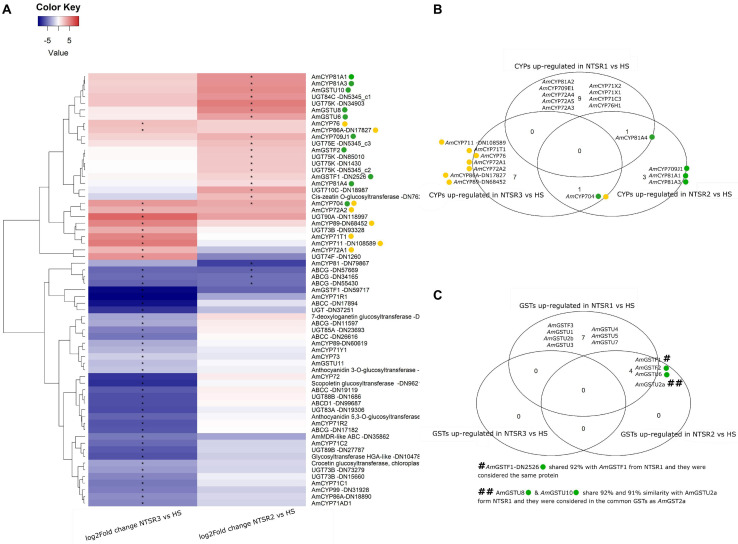
**(A)** Heatmap of the log_2_ of the fold change of the xenome genes (including CYPs, GSTs, UGTs, and ABCs) of the NTSR2 vs. HS and NTSR3 vs. HS. Star in represent the FDR ≤ 0.05. Clustering dendrogram of the samples based on the log_2_FC was represented at the left of the heatmap. **(B)** Venn diagram representing the CYPs upregulated (log_2_FC ≥ 1 and FDR ≤ 0.05) in NTSR1, NTSR2, and NTSR3 vs. HS. **(C)** Venn diagram representing the GSTs upregulated (log_2_FC ≥ 1 and FDR ≤ 0.05) in NTSR1, NTSR2, and NTSR3 vs. HS. CYPs and GSTs upregulated in NTSR3 and NTSR2 were highlighted using yellow and green circles, respectively, in the heatmap and the Venn diagrams. HS, sensitive population; NTSR1, Peldon in-field population with broad-ranging resistance to post-emergence herbicides; NTSR2, derived from HS and selected for resistance to pendimethalin; NTSR3, derived from HS and selected for resistance to fenoxaprop-*P*-ethyl; FDR, false discovery rate; CYPs, cytochrome P450s; GSTs, glutathione transferases.

With their known involvement in herbicide resistance linked to enhance metabolism, it was of immediate interest to establish how the genes encoding the different families of proteins involved in xenobiotic detoxification were expressed in the NTSR2 and NTSR3 plants. As a primary analysis, the differential expression of phase 1 (CYPs), phase 2 (GSTs and UGTs), and phase 3 (ABC transporters) gene families was compared. A total of 25 CYPs, 7 GSTs, 24 UGTs, and 10 ABCs were differentially expressed in the NTSR plants relative to the HS progenitor population, with a heatmap showing the log_2_ fold change of each xenome gene for each population (Figure 5). The sequences of the respective proteins (Supplementary Table S4) were subject to BLASTp analysis in order to identify the family members. In total, CYPs from 11 families were identified (704, 709, 71, 711, 72, 73, 76, 81, 86, 89, and 99). Relative to the HS plants, the largest number of CYP DEGs (19) was determined in the NTSR3 plants, with 8 upregulated and 11 downregulated. These DEGs belonged to all CYP families, except family 81. In contrast, only six CYPs, belonging to families 704, 709, and 81, were differentially expressed in the NTSR2 population. The CYP 81, 71, 72, and 709 families were of particular interest, due to their known connection with herbicide metabolism in crops and EMR in NTSR1 Peldon black-grass. In each case, the respective genes were assigned identities based on their phylogenies (data not shown). Based on this analysis, *Am*CYP81A4 was upregulated in both the NTSR1 and NTSR2 populations, with the orthologous (93% identical), *Am*CYP81A2 (identified in NTSR1), and *Am*CYP81A3 (identified in NTSR2) also co-enhanced. In contrast, no CYPs from these families were differentially expressed in the NTSR3 population relative to the HS population.

Seven GST DEGs were identified, with two downregulated tau (U) genes in NTSR3 (*Am*GSTF1 DN5917 and *Am*GSTU11) and five upregulated in NTSR2 (*Am*GSTF1, *Am*GSTF2, *Am*GSTU10, *Am*GSTU2, and *Am*GSTU6). In order to investigate the potential common mechanisms of detoxification between the NTSR1 and NTSR2 populations, all the GSTUs previously identified in resistant black-grass were compared (Nandula et al., 2019). *Am*GSTU6, *Am*GSTU8, and *Am*GSTU10 showed elevated expression in the NTSR2 plants. *Am*GSTU8 and *Am*GSTU10 were of particular interest, as they were both very similar (91 and 92%) to an enzyme previously termed *Am*GSTU2a, which was upregulated in the NTSR1 Peldon population and showed activity toward several herbicides (Nandula et al., 2019). In contrast, the only GSTU identified in the NTSR3 plants named *Am*GSTU11, which was an ortholog of the bronze 2 gene in maize (Marrs et al., 1995) involved in anthocyanin deposition in the vacuole, showed a lower level of expression than determined in the HS population. The previous studies in NTSR1 Peldon had also identified two phi (F) class enzymes, *Am*GSTF1 and *Am*GSTF2, as being upregulated relative to HS, with *Am*GSTF1 isoenzymes known to be encoded by multiple gene variants (Cummins et al., 2013). Similarly, the current study identified two variants termed *Am*GSTF1-DN2526 and *Am*GSTF1-DN59717. *Am*GSTF1-DN2526 was upregulated in NTSR2 but not in NTSR3, while *Am*GSTF1-DN59717 expression was depressed in both populations. Four coding sequences of the *Am*GSTF1-DN2526 were retrieved, with the sequences being shorter than the four isoforms previously identified (Cummins et al., 2013). Three of these were 99, 99, and 83%, similar, respectively, to *Am*GSTF1a and b, with the other ORF showing additional 97% similarity to *Am*GSTF1d. In addition to *Am*GSTF1, *Am*GSTF2 was also identified, though its expression was only enhanced in the NTSR2 plants. This protein was 100% similar with the *Am*GSTF2 previously identified in resistant NTSR1 black-grass (Tétard-Jones et al., 2018).

With respect to other classes of xenome genes, UGTs from the families 710C, 73B, 74F, 75E, 75K, 83A, 84C, 85A, 88B, 89B, and 90A were differentially expressed. Relative to the HS plants, a total of 13 UGTs were downregulated and three upregulated in NTSR3 (*Am*UGT73B-DN93328, *Am*UGT74F-DN1260, and *Am*UGT90A-DN118977), with 9 UGTs (*Am*UGT710C-DN18987, *Am*UGT75E-DN5345_c3, four *Am*UGT75K, *Am*UGT84C-DN5345_c1, and a *cis*-zeatin *o*-glycosyltransferase-DN762) upregulated in NTSR2. Of the 10 ABC proteins identified, 4 belonged to the family C, 1 to the D, 5 to the G, and 1 to the multidrug resistance (MDR) ABC; all were downregulated in NTSR3, while only 3 family G ABCs were downregulated in NTSR2.

Attention was then focused on the NTSR biomarkers that had been clearly identified in previous proteomic studies (Tétard-Jones et al., 2018). Of the eight biomarkers, only OPR-1 and NADPH quinone oxidoreductase 1 were determined as DEGs in NTSR2 (log_2_FC = 4.5) and none in NTSR3 (Table 1). Based on the relative enhanced expression of the proteins, only OPR1 was determined as being strongly induced in the transcriptome of the NTSR2 plants (Table 1).

**TABLE 1:** Expression of five out of the eight molecular markers identified by Tétard-Jones et al. (2018) that are not part of the xenome.

	**Global expression**				**FC of protein abundance**	
	**in this study**				**as reported by Tétard-Jones et al. (2018)**	
	**log_2_FC NTSR2 vs. HS**	**FDR NTSR2 vs. HS**	**log_2_FC NTSR3 vs. HS**	**FDR NTSR3 vs. HS**	**NTSR2 vs. HS**	**NTSR3 vs. HS**
*Am* 12-oxophytodienoate reductase 1 (OPR-1)	4.5	1.20E-43	−0.8	1.0	4.1	1.1
*Am* D-3-phosphoglycerate dehydrogenase 1	1.6	NA	−2.2	NA	3.2	1.2
*Am* NADPH quinone oxidoreductase 1	4.5	0.1	−2.1	1.0	1.7	1.1
*Am* stem-specific protein TSJT1	0.5	1.0	−0.4	1.0	1.7	1.1
*Am* NAD-dependent epimerase/dehydratase	0.2	1.0	0.1	1.0	1.7	1.1

*The table includes the protein amount in each population in order to compare the transcriptome with the proteomic data. HS, herbicide-sensitive population; NTSR2, derived from HS and selected for resistance to pendimethalin; NTSR3, derived from HS and selected for resistance to fenoxaprop-P-ethyl; FC, fold change; FDR, false discovery rate; Am, Alopecurus myosuroides.*

### Weighted Gene Co-expression Network Analysis of NTSR2 and NTSR3 Populations

**FIGURE 6:**
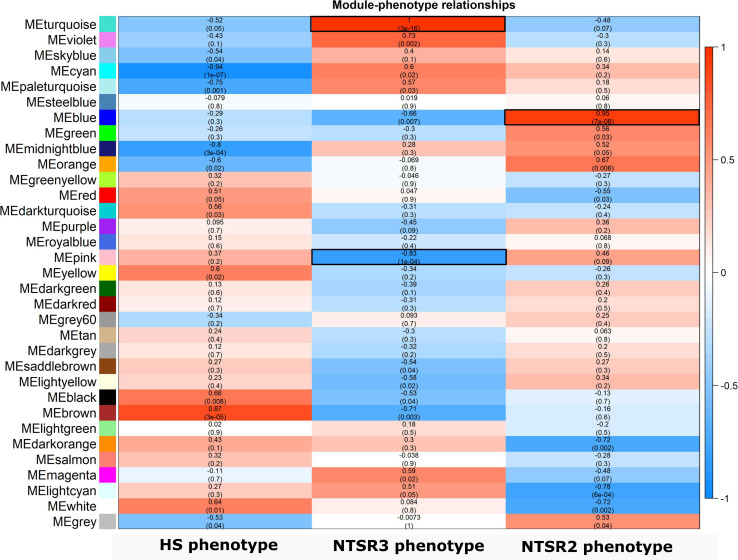
Heatmap of the bicor correlation between the eigengenes of each WGCNA modules. Each row represents a module eigengene and each column a phenotype determined by the resistant or sensitive to the different herbicides. *R*-value and *P*-values of the bicor correlation between the eigengenes and the phenotypes are indicated in each square. In red, positive correlations; and in blue, negative correlations. HS, sensitive population; NTSR3, derived from HS and selected for resistance to fenoxaprop-*P*-ethyl; NTSR2, derived from HS and selected for resistance to pendimethalin; WGCNA, weighted gene co-expression network analysis.

In contrast to NTSR2, the large-scale downregulation of detoxification-related transcripts in the NTSR3 population further suggested that mechanisms other than EMR were responsible for resistance in these plants. To couple changes in transcript expression to resistance traits in the NTSR2 and NTSR3 plants, a WGCNA was performed on a total of 54,728 unigenes (variance cut-off 0.8), allowing for genes showing similar expression patterns to be grouped into modules, giving potential insight into their functional relatedness. This analysis grouped transcripts into 33 modules, each assigned a specific color, which correlated with putative resistance traits in the NTSR2 and NTSR3 populations (Figure 6). Three unique modules (turquoise, pink, and blue) that showed an absolute Pearson correlation coefficient (*r*) > 0.8, *P* ≤ 0.05, were of particular relevance and were further analyzed. On the basis of positive correlation, the turquoise module had the highest module–trait relationship with the NTSR3 phenotype (*r* = 1; *P* = 3E-16), while the blue module related to the NTSR2 phenotype (*r* = 0.95; *P* = 7E-08; Figure 6). In contrast, the pink module showed a high negative Pearson correlation (*r* = -0.83 and *P*-value = 1E-04) with the NTSR3 phenotype.

To connect transcript expression to phenotypic traits, HUB genes were retrieved on the turquoise, pink, and blue modules. Highly connected HUB genes typically influence the expression of other genes and may be causal factors in phenotypic traits. To identify HUB genes, the correlation between the GS and the MM was plotted (Supplementary Figures S1E–G), with the GS representing the absolute value of the correlation between each unigene in the analysis and the phenotype, while the MM measures how connected a given gene is to other genes in the same module. The three selected modules showed a high correlation between the GS and MM, indicating that the majority of the genes in these modules are linked to traits that define each respective population (Supplementary Figure S1). Next, the criteria of GS ≥ 0.2 and MM ≥ 0.8 applied to identify HUB genes. A total of 2,503, 263, and 1,053 HUB genes were identified in the turquoise, pink, and blue modules, respectively, with the annotations and individual GSs and MMs listed in Supplementary Table S5.

### HUB Gene Analysis Reveals Fundamental Differences in Resistance Mechanisms in NTSR2 and NTSR3 Plants

**FIGURE 7:**
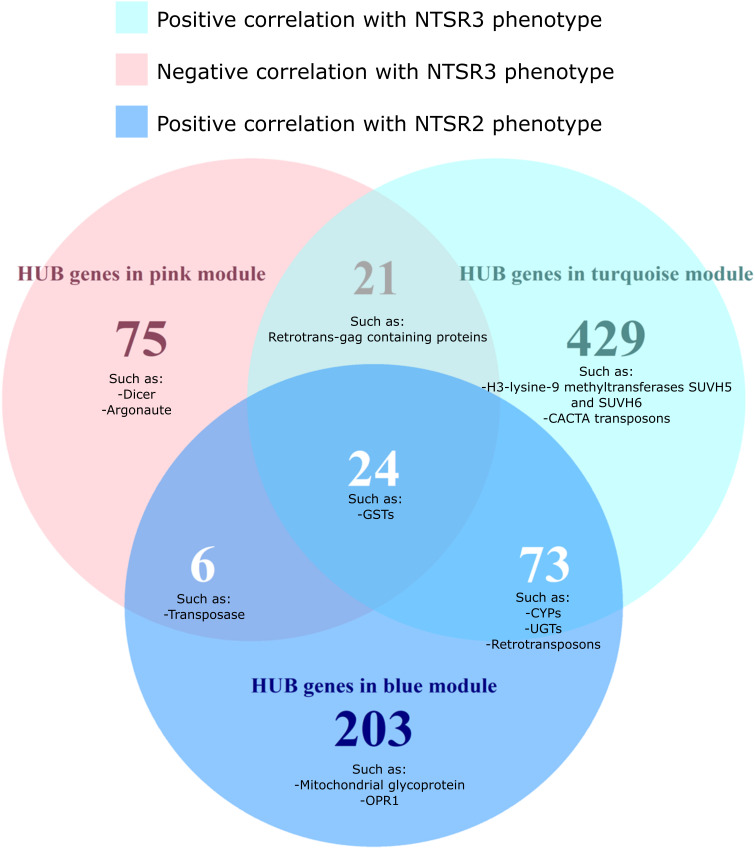
Venn diagram of the differentially expressed HUB genes of the turquoise, pink, and blue modules grouped by the AHRD annotations. The number on each circle represents the number of different AHRD annotations. Human readable description of the common HUB genes between the turquoise (positively correlated with the NTSR3 population, pink (negatively correlated with the NTSR3 population), and blue (positively correlated the NTSR2 population) modules. HS, sensitive population; NTSR3, derived from HS and selected for resistance to fenoxaprop-*P*-ethyl; NTSR2, derived from HS and selected for resistance to pendimethalin.

To identify the function of genes that are important for the two NTSR traits, HUB genes were identified for each module and grouped by function using AHRD (human readable) annotation (Supplementary Table S6). In total, 547, 126, and 306 different annotations, respectively, were found in the HUB gene list of the turquoise, pink, and blue modules (Supplementary Table S6). A Venn diagram of the common and different annotations of HUB genes in each module showed that 24 different annotations were shared among the HUB genes of the three modules, including GSTs disease resistance proteins and GSTs (Figure 7 and Supplementary Table S6).

While the NTSR2 and NTSR3 populations showed clearly distinct phenotypes, 73 annotations were shared between the respective turquoise and blue modules. These annotations included xenome genes, such as CYPs and glycosyltransferases, as well as transposable elements (TEs), including retrovirus-related polyproteins from transposon TNT1-94 and Ty3-gypsy retrotransposons. Other transcripts corresponding to the proteins Dicer, Argonaute, SUVH5, SUVH6, and members of the CACTA transposon En/Spm subclass were only found in the HUB list of the turquoise and pink modules exclusively associated with the NTSR3 phenotype (Figure 7).

Different xenome genes were identified in each module, turquoise (7 CYPs, 3 GSTs, 8 UGTs, and 4 ABCs), pink (1 GST and 1 ABC), and blue (5 CYPs, 1 GSTs, 12 UGTs, and 4 ABCs) (Supplementary Figure S2) and compared against the xenome genes identified by DEG analysis. Despite there being 32 CYPs and GSTs DEGs identified in NTSR3 compare with the HS population, only *Am*CYP72A1 was also identified in the HUB list of the turquoise module (Supplementary Figure S2A). The other 10 CYPs were not differentially expressed but, according to the WGCNA, have a role in determining the NTSR3 phenotype. Interestingly, AmGSTF_2526_c0, which was 60% similar to *Am*GSTF1 previously identified in NTSR1 Peldon, was identified as a HUB gene in the pink module, which negatively correlates with the NTSR3 phenotype. In NTSR2, out of the 11 upregulated or downregulated CYPs and GSTs, only *Am*CYP81A4 and *Am*GSTU10 were identified by both analyses (Supplementary Figure S2B), highlighting them as potential functional detoxification biomarkers. Within the additional biomarkers identified by Tétard-Jones et al. (2018), only OPR1 was part of the HUB gene list of the blue module correlating with the NTSR2 phenotype, being highly upregulated in this population (Figure 7). The other biomarker proteins were not identified on the HUB gene list, as they did not pass the cut-off defined in this study.

A GO enrichment was then performed on the HUB gene list of the three selected modules. A total of 414, 68, and 184 unigenes within the HUB genes of the turquoise, pink, and blue modules were annotated to GO terms, respectively. GOs with a *P*-value of the Fisher test ≤ 0.05 are shown (Table 2), with the full list of genes associated with each term presented in Supplementary Table S7. The greatest enrichment in GO terms in the turquoise module positively correlating with the NTSR3 phenotype was genes involved in biological process such as transposition and ADP binding (Table 2). The enrichment of genes such as those encoding the CACTA protein, which are involved in transposition, coincided with the identification of this type of TE in the same module (Figure 7). Genes with molecular functions linked to ADP binding were also enriched in the turquoise module, including disease resistance proteins and NB-ARC (nucleotide-binding adaptor shared by APAF-1, resistance proteins, and CED-4), which are required for activating plant immunity. KEGG enrichment of these modules also showed an association with the flavone and flavonol biosynthesis pathway (Supplementary Table S8). To further investigate the potential significance of these HUB genes, the change in their expression was compared with that determined in other plants exposed to different treatments using Genevestigator. When the HUB genes representing 16% of the total present in the turquoise module were compared against the different RNA-Seq experiments performed in wheat, barley, and maize, the 400 most significant genes were linked to induction by biotic stresses caused by infection with the fungal pathogen *Fusarium graminearum*, or with osmotic stress (Supplementary Table S9).

**TABLE 2:** Gene Ontology (GO) enrichment (biological process, molecular function, and cellular component) of the HUB gene list of the turquoise, pink, and blue WGCNA modules highly correlated with the NTSR2 and NTSR3 phenotypes.

	**GO term**	**% significant genes**	***P*-value**
Turquoise module (HUB genes). Positive correlation with NTSR3 population phenotype	Protein-containing complex assembly (GO:0065003)	15.79	0.007
	Guanosine tetraphosphate metabolic process (GO:0015969)	15.38	0.038
	Transposition (GO:0032196)	14.29	0.043
	ADP binding (GO:0043531)	2.92	0.014
Pink module (HUB genes). Negative correlation with the NTSR3 population phenotype	Calmodulin binding (GO:0005516)	5.00	0.036
	Helicase activity (GO:0004386)	4.76	0.038
Blue module (HUB genes). Positive correlation with NTSR2 population phenotype	Hydrolase activity, acting on carbon–nitrogen (but not peptide) bonds, in linear amides (GO:0016811)	33.33	0.00004
	Calcium ion binding (GO:0005509)	3.66	0.027
	Mitochondrial matrix (GO:0005759)	20.00	0.044
	SAGA-type complex (GO:0070461)	20.00	0.044
	Mitotic sister chromatid cohesion (GO:0007064)	20.00	0.014
	Organelle organization (GO:0006996)	6.67	0.018

*Number of significant genes was calculated by dividing the number of significant genes in the dataset by the total of genes annotated to that GO term. WGCNA, weighted gene co-expression network analysis.*

The most enriched GO terms in blue module linked to the NTSR2 phenotype were genes encoding proteins involved in calcium binding (EF-hand domain-containing protein) organelle function (mitochondrial glycoprotein) and zinc ribbon domain-containing proteins (Table 2 and Supplementary Table S6). From this analysis, it was perhaps surprising that GO terms such as GO:0098754 linked to detoxification were not enriched in any module, especially in the module linked to NTSR2, even though several xenome genes were identified as HUB genes (Supplementary Figure S2). However, KEGG enrichment did show a link between the blue module and the caprolactam degradation pathway, which is linked to xenobiotic metabolism (Supplementary Table S7). Genevestigator analysis of the HUB genes from the blue module against other crop datasets showed multiple links to genes induced by biotic and abiotic stresses. In comparison with wheat, 22 genes of the blue module resembled responses to cold stress and infection by *Puccinia striiformis* (causal agent of yellow rust), while the expression of 375 genes of the same module identified an orthologous response in barley to infection by *F. graminearum*. On comparison with maize, the expression of 334 genes of the blue module resembled the response to submergence stress (Supplementary Table S9).

## Discussion

In this study, we have utilized WGCNA to search for differences in NTSR mechanisms arising in two populations of black-grass, each selected from a common ancestor by repeated treatment with different herbicides. This study did not include inducible resistance mechanisms triggered after herbicide treatment but instead focused on constitutive NTSR mechanisms (Cummins et al., 2009; Délye, 2013). As compared with HS parent plants, the NTSR2 population, selected through 8 years of consecutive treatment with the preemergence herbicide pendimethalin was shown to have an enhanced ability to detoxify chlorotoluron. As such, the NTSR2 trait showed many similarities to the EMR demonstrated in the classic NTSR1 population Peldon. Network analysis demonstrated a strongly positively correlated group of genes; the blue module linked to the NTSR2 phenotype that included CYPs and GSTs likely to function in the detoxification of pendimethalin and other selective herbicides. In contrast, in the NTSR3 plants selected for resistance to fenoxaprop-*P*-ethyl over six consecutive years, detoxification genes were not central to the resistance trait, with a distinct turquoise module of genes more functionally similar to patterns of gene expression linked to responses to pathogen and drought stress in cereals being observed.

In examining the role of xenome genes in NTSR in untreated black-grass plants, a large number of CYPs, GSTs, UGTs, and ABCs were identified as DEGs. In the NTSR2 population, relative to HS plants, multiple CYPs, GSTs, and UGTs were upregulated, while surprisingly, the ABC transporters were downregulated. In contrast, in the NTSR3 plants, the majority of xenome genes were downregulated irrespective of function. Based on their known role in the primary detoxification of herbicides, the CYP and GST superfamilies were of particular interest in the NTSR2 resistance mechanism. In wild grasses, CYPs are involved in the initial metabolism and inactivation of herbicides acting on ACCase (Kreuz and Fonné-Pfister, 1992; Ahmad-Hamdani et al., 2013), ALS (Iwakami et al., 2014a, 2019), and other modes of action (Evans et al., 2017). The data presented here point to the importance of the CYP81A family in NTSR in the NTSR2 population. *Am*CYP81A4 was enhanced in both NTSR2 and NTSR1 Peldon populations and was among the HUB genes of the WGCNA blue module that correlated with the respective resistance phenotype. A growing body of evidence now points to a central role for members of the CYP81A family in EMR in wild grasses (Iwakami et al., 2014a, 2019). Other CYPs upregulated in the NTSR2 plants included members of families 704 and 709, though WGCNA analysis did not point to these as being essential HUB genes.

Intriguingly, other CYP family members were identified as upregulated DEGs in the NTSR3 plants that did not evidence enhanced herbicide metabolism, including CYP711, a CYP71, and two CYP72s, as well as a CYP76, a CYP86, and a CYP89. One of these genes, *Am*CYP72A1, was identified as an NTSR3 HUB gene. Since the NTSR3 phenotype does not involve EMR, this would suggest that these induced CYPs must be playing roles in endogenous metabolism rather than in xenobiotic detoxification. Intriguingly, many related genes have also been reported to be upregulated in other NTSR weeds. These include *Ep*CYP71AK2 and *Ep*CYP72A254 in bispyribac-resistant *Echinochloa phyllopogon* (Iwakami et al., 2014b); members of families 71, 72, and 89 in *Lolium rigidum* populations resistant to diclofop and to pyroxsulam (Gaines et al., 2014; Duhoux et al., 2017); and *Ma*CYP76C1 and *Ma*CYP86B1 in *Myosoton aquaticum*-resistant plants (Liu et al., 2018). In addition, related genes are frequently induced by herbicide exposure, including families 72 and 81 induced in *Lolium* after treatment with iodosulfuron and mesosulfuron (Duhoux et al., 2017), *Ae*CYP71A4 in *Alopecurus aequalis* in response to fenoxaprop-*P*-ethyl and mesosulfuron-methyl (Zhao et al., 2017, 2019), and multiple CYP72As elevated in *Alopecurus japonicus* by fenoxaprop-*P*-ethyl (Chen et al., 2018). It therefore seems probable that a large number of CYPs enhanced by herbicides and/or NTSR in weeds relate to endogenous inducible stress responses to foreign compounds. As an example, unlike the CYP81As, the large CYP72A family is widely distributed in higher plants (Hamberger and Bak, 2013), assuming roles involved in the biotransformation of mono- and tri-terpenes (Leveau et al., 2019) and gibberellins (He et al., 2019). However, in cereals, some CYP72As are also known to be involved in herbicide metabolism, with *Os*CYP72A31 from *Oryza sativa* acting on bensulfuron-methyl (Ohno et al., 1991) and *Os*CYP72A18 catalyzing the hydroxylation of pelargonic acid (Imaishi and Matumoto, 2007).

On comparing CYP genes that were upregulated in more than one resistant population, *Am*CYP704, a member of a CYP family not previously linked to herbicide resistance in weeds was found to be upregulated in both the NTSR2 and NTSR3 plants. Previous transcriptome studies in NTSR1 Peldon identified several abundant CYPs, including *Am*CYP71X1, *Am*CYP71C3 and *Am*CYP71X2, which were not elevated in the NTSR2 plants (Tétard-Jones et al., 2018). The presence of these additional CYPs in Peldon may help explain the subtle differences in the resistance traits between the NTSR1 and NTSR2 black-grass. Significantly, several members of family 71 have known roles in herbicide detoxification, notably *Ta*CYP71C6v1 from wheat, which is able to metabolize sulfonylurea herbicides when expressed in yeast (Xiang et al., 2006).

GSTs, particularly *Am*GSTF1, have also been shown to have critical roles in NTSR in black-grass and other wild grasses (Cummins et al., 2013). In black-grass, *Am*GSTF1 is encoded by four genes, termed *Am*GSTF1a, b, c, and d, though only the 2c and 2d isoforms were detected in the previous proteome analysis of NTSR plants (Tétard-Jones et al., 2018). From the transcriptome data in the current study, two *Am*GSTF1 variants were identified, though the transcripts were too short to accurately determine their identities. The ORF of *Am*GSTF-DN2526 was the most similar to the 2a or 2b isoforms. Transcripts encoding this variant were highly upregulated in the NTSR2 but not in NTSR3 plants. Given the apparently critical role of *Am*GSTF1 in NTSR in black-grass, this complex regulation of its isoenzymes is intriguing, though to date no functional significance has been attributed to the respective protein variants. Certainly, the results of the transcriptomic study reported here suggest that the transcriptional regulation of *Am*GSTF1 cannot account for its quantitative regulatory role recently confirmed in NTSR (Comont et al., 2020). While *Am*GSTF1 protein expression was elevated in both the NTSR2 and NTSR3 populations, enhanced levels of the respective transcripts were only determined in the NTSR2 plants. In addition, HUB analysis identified a sequence showing only 60% similarity to the *Am*GSTF1 transcript in the pink module, and it was therefore questionable whether *Am*GSTF1 mRNAs play a regulatory role in NTSR. Instead, out data suggested that the known regulatory function of the *Am*GSTF1 protein in NTSR must be regulated transcriptionally or post-transcriptionally.

In addition to *Am*GSTF1, the additional phi class *Am*GSTF2, which shares 100% similarity with the one previously identified by Tétard-Jones et al. (2018), was also highly upregulated in the NTSR2 but not in NTSR3 plants. As previously reported by Nandula et al. (2019), this *Am*GSTF2 shared high a similarity with other GSTFs induced by safeners in cereals, including *Zm*GSTF4 in maize and *Ta*GSTF3 in wheat, which are able to both metabolize chloracetanilide herbicides and act as glutathione peroxidases (Cummins et al., 2003).

In total, four GSTUs were differentially expressed in NTSR black-grass, with three of them being upregulated in NTSR2. Of these, *Am*GSTU10 was identified as a HUB gene in the associated blue module, further confirming its role in resistance (Tétard-Jones et al., 2018). *Am*GSTU10 shared a high similarity with the enzyme termed *Am*GSTU2a previously identified in NTSR1 Peldon, which was active toward the herbicides fenoxaprop-*P*-ethyl and metolachlor (Nandula et al., 2019). It would seem most likely that *Am*GSTU10 and *Am*GSTU2a are derived from gene duplication. Concerning other NTSR mechanisms linked to the xenome, proteomic studies had identified OPR1 as a resistance biomarker (Tétard-Jones et al., 2018). In the current study, OPR1 was also identified as a HUB gene in the blue module correlated with the NTSR2 resistance phenotype, consistent with the importance of this gene in resistance.

In our previous transcriptome and proteome studies with the Peldon NTSR1 population, several similarities were determined between NTSR in black-grass and MDR in humans (Cummins et al., 2013; Tétard-Jones et al., 2018). The current study further confirmed a link between MDR mechanisms and changes in gene expression associated with NTSR2, which most closely resembles NTSR1. Intriguingly, genes enriched in the blue NTSR2 module, such as zinc ribbon domain-containing proteins, EF-hand and calcium ion binding proteins, and *P*-glycoproteins, are also associated with MDR in cancer cells (Hong et al., 2005; Shen et al., 2012). Of relevance to xenobiotic detoxification, *P*-glycoproteins are known to be involved in the membrane transport and export of cytotoxic drugs, being associated with regulatory zinc ribbon domain-containing proteins (Hong et al., 2005; Sulová et al., 2009).

Increased expression of transcripts involved in stress responses, e.g., oxidative stress, has previously been identified in NTSR1 Peldon plants (Tétard-Jones et al., 2018). Genevestigator analysis revealed that HUB genes associated with both the NTSR2 and NTSR3 phenotypes corresponded with transcripts linked to responses to biotic (infection with *Fusarium graminearum* or *Puccinia striiformis*) and abiotic (cold and osmotic) stresses in cereals. Together, these results suggest that plant responses to adverse environmental conditions are integrally linked to NTSR-based herbicide resistance. In support of this hypothesis, a link between TEs linked to stress tolerance and NTSR was determined. TEs were enriched in the modules (turquoise and pink) highly correlated with the NTSR3 population, notably CACTA TEs and retrotransposon gag proteins (Kunze et al., 1997; Todorovska, 2007). TEs were also found in the NTSR2 population, including the long terminal repeat (LTR) retrotransposon and the *Pseudoviridae* Ty1-copia retrotransposon (Kumar and Bennetzen, 1999). TEs are a catalyst for genetic variation and are known to be activated by environmental stress (Takeda et al., 1999; Kimura et al., 2001; Scheid et al., 2010; Woodrow et al., 2011), playing critical roles in the adaptation of plants to abiotic stress (Naito et al., 2009; Scheid et al., 2010; Sigman and Slotkin, 2015). Therefore, we speculate that TEs could play a key role in the evolution of NTSR in the black-grass populations, with each phenotype associated with the recruitment of distinct groups of TEs.

In conclusion, WGCNA is a useful tool to identify important and previously unknown gene networks mediating NTSR using transcriptome datasets. In particular, the potential connection between NTSR and plant responses to (a)biotic stress mechanisms has been identified in this study. This current finding starts to shed light on the complexity of NTSR and to provide important basic information to interrogate the evolutionary route of NTSR in black-grass and other grass weeds. In addition, it will now be of interest to study the changes in the transcriptomes of these NTSR populations that result from herbicide treatment to determine chemically inducible as well as latent resistance mechanisms in black-grass.

## Data Availability Statement

The original contributions presented in the study are publicly available. This data can be found here: GEO repository (GSE162422).

## Author Contributions

RE conceived the study and was in charge of funding acquisition and supervision. NO cultivated and provided the plant material and performed the metabolism analysis and the ELISA. MB-H helped in the design, data curation, and revision. AW helped in the cultivation of the plants and the extraction of the RNA. AG-C helped in the extraction of the RNA and its quality control, identified the DEGs, and performed the phylogeny trees that determined the nomenclature of the CYPs. SF-O analyzed the RNA-seq, conducted the gene correlation network, the GO and KEGG enrichment, and the Genevestigator analysis and drafted the manuscript. All authors participated in the revisions and approved the final manuscript.

## Conflict of Interest

The authors declare that the research was conducted in the absence of any commercial or financial relationships that could be construed as a potential conflict of interest.

## Footnotes

1 https://github.com/groupschoof/AHRD

2 https://www.araport.org

3 ftp://ftp.ebi.ac.uk/pub/databases/GO/goa/UNIPROT/goa_uniprot_all.gaf.gz

4 ftp://ftp.ebi.ac.uk/pub/databases/interpro/61.0/interpro.xml.gz

5 https://www.ncbi.nlm.nih.gov/

6 https://blast.ncbi.nlm.nih.gov/

7 https://horvath.genetics.ucla.edu/html/CoexpressionNetwork/Rpackages/WGCNA/faq.html

8 https://horvath.genetics.ucla.edu/html/CoexpressionNetwork/R.packages/WGCNA/faq.html
