# Supplementary material for: Non-target Site Herbicide Resistance Is Conferred by Two Distinct Mechanisms in Black-Grass (Alopecurus myosuroides)
Source: Front Plant Sci. 2021 Mar 3;12:636652. doi: 10.3389/fpls.2021.636652 (PMC7966817; doi:10.3389/fpls.2021.636652)
Supplement: Supplementary file 1 [file Data_Sheet_1.docx]

Supplementary Material

# Supplementary Data

## Illumina sequencing

The cDNA library construction, the Illumina sequencing and *de nov*o transcriptome assembly were performed by using the services of Earlham Institute (Norwich, UK). High quality libraries were prepared with the NEBNext Ultra™ RNA Library Prep Kit for Illumina (New England BioLabs (NEB), Ipswich, USA) using manufacturer’s instructions. The process was performed on the Perkin Elmer Sciclone G3 workstation area (PerkinElmer (Madrid, Spain). One ug of the poly (A) mRNA was purified from the total RNA using Oligo d(T)25 paramagnetics beads and the mRNA Magnetic Isolation Module (NEB). Then, the mRNA of interest was fragmented by incubation at high temperature, and hybridised to random hexamers primers for reverse transcription. Initially, first strand cDNA synthesis was performed. Afterwards, the second strand synthesis process removed the RNA template and generated ds cDNA. Uraciles were only incorporated in the second strand., to retain directionality. The double-stranded DNA molecules were then cleaned-up and end-repaired in order to create blunt, phosphorylated ends. A single adenine was added to the 3-primer end to allow highly efficient ligation using the thymine end of the Hairpin loop-shaped NEBNext adaptors. USER™ enzyme (a combination of UDG and Endo VIII) was used to remove the strand containing the uraciles and to open the hairpin loop of the adapter that contained a uracile, which made it available as a substrate for a PCR. The products were then cleaned up again using a beads-based purification to remove most of the un-ligated adaptors and a PCR amplification with a Q5 High Fidelity DNA polymerase was performed following manufacturer’s instructions (30 s at 98°C, followed by 10 cycles of: 10 s at 98°C, 75 s at 65°C and 5 min at 65°C, with a final hold at 4°C). Barcodes were incorporated by using NEBNext Multiplex Oligos for Illumina® (96 Unique Dual Index Primer Pairs, NEB) during the PCR to allow multiplexing. cDNA strand was then sequenced.

The insert size of the libraries was verified by running an aliquot of the DNA library on a LabChip® GX Touch™ Nucleic Acid Analyzer (PerkinElmer) with the DNA High Sensitivity Reagent Kit (PerkinElmer). The concentration was also determined by using Qubit™ RNA High Sensitivity Assay Kit (ThermoFisher Scientific, Cramlington, UK) on the Qubit 2.0 Fluorometer (ThermoFisher Scientific). Additionally, these libraries were pooled and checked by qPCR to ensure the presence of the sequencing adapters. The library pool was diluted to 0.65 nM using 10 mM Tris at pH 8.0, in a final volume of 18 µl and 1% phiX control v3 (Illumina, Cambridge, UK) was spiked to the diluted library. The library was denatured by adding 4 µl of 0.2 N NaOH and incubating at room temperature for 8 min. Afterwards 5 µl of 400 mM Tris at pH 8.0 were added to neutralise the reaction. A master mix of EPX1, EPX2, and EPX3 from Illumina’s Xp 2-lane kit (Illumina) was prepared and 63 µl were added to the denatured library pool (final volume of 90 µl at 130 pM) that were then loaded onto the NovaSeq 6000 (Illumina) along with NovaSeq 6000 SP cluster cartridge, buffer cartridge, and 300 cycle SBS cartridge. The sequencing was performed on a NovaSeq 6000 System (Illumina) with NovaSeq Control Software (NVCS) v1.6 and basecalling was performed using Real-Time Analysis (RTA) v3.4.4. The instrument was set up to sequence 150 bp paired-end reads. The raw files obtained from the NovaSeq 6000 were demultiplexed and transformed to raw reads and stored in FASTQ files using bcl2fastq2 Conversion Software v2.20 (Illumina).

## Cytochrome P450: Family and subfamilies classification

Cytochrome p450 is a super family of proteins categorized into clans, families and subfamilies.

Members of the four families 81, 71, 709 and 72 were investigated in more detail to establish their subfamily membership. The subfamily classification was achieved by performing a separate phylogenetic analysis using as a framework sequences from wheat (Li and Wei, 2020). To this end, sequences were manipulated using SEAVIEW software version 4 .6.4 (Gouy et al. 2010). Sequences were aligned with Muscle (Edgar 2004) and subsequent trimmed using trimAI software with defaults settings v.1.3 (Capella-Gutiérrez et al. 2009) accessed through the webserver Phylemon 2 (Sánchez et al. 2011). The trimmed alignment was used to infer maximum likelihood phylogenies with the IQ-TREE software using the online server (Trifinopoulos et al., 2016). The automatic mode was used to select the best fitting model for each alignment and 1000 ultrafast bootstrap support values calculated for branch support.

Those xenoma genes with very short sequences were not subjected to phylogenetic analysis and were no classified into subfamilies.

# Supplementary Figures and Tables

## Supplementary Figures





**Supplementary Figure 1.** **A.** Assessment of scale-free fit index (2 to 20) for various soft-thresholding powers (β). **B** Assessment of mean connectivity for various soft-thresholding powers (β) (2 to 20). **C**. Clustering dendrogram of the weighted gene co-expression network analysis (WGCNA). Colors represent the different WGCNA modules for the RNA Seq data. D. Clustering dendrogram of the samples based on the Euclidean distance of the phenotype heatmap. In red positively correlated sample to phenotype and in white negatively correlated samples to phenotypes. Scatter plots of gene significance (GS) and the module membership (MM) of the genes identified in the turquoise (**E**), pink (**F**) and blue (**G**) modules.

**
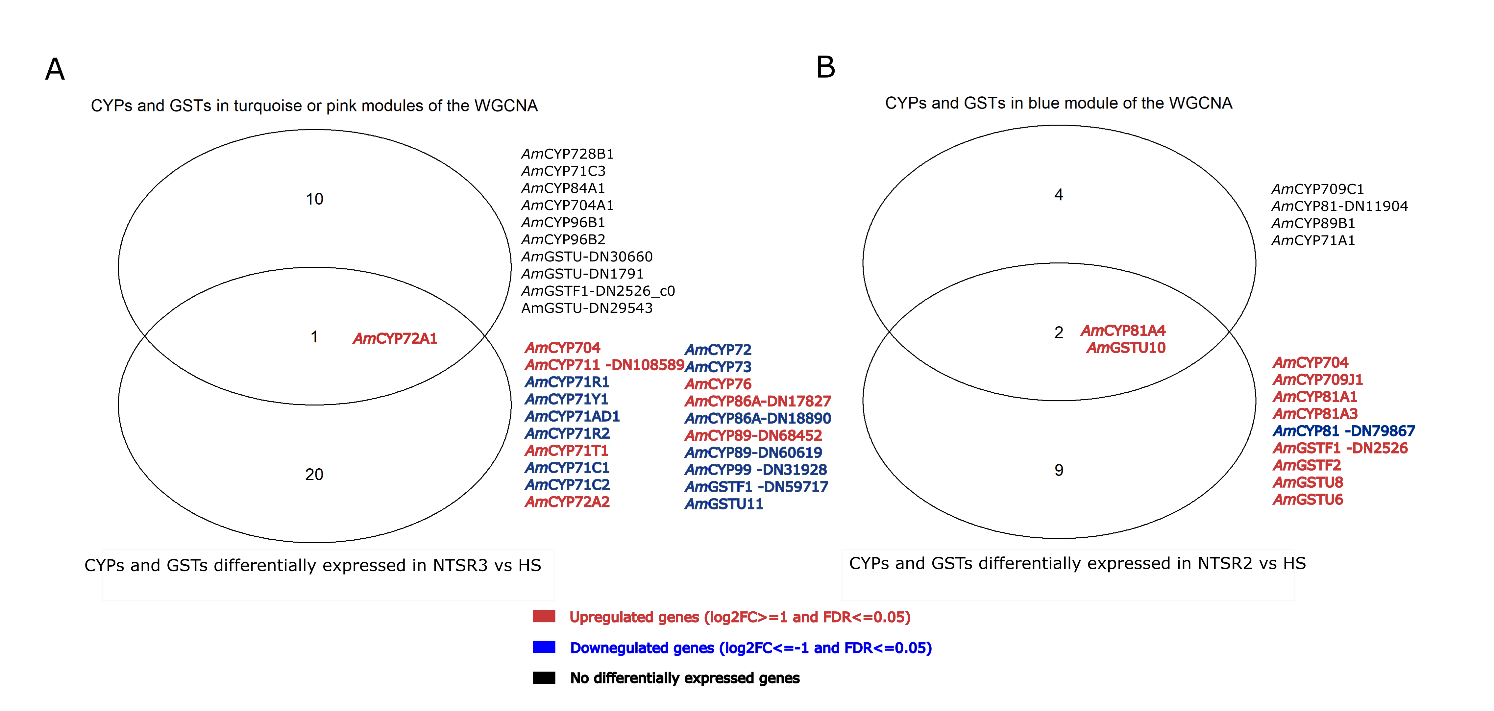
**

**Supplementary Figure 2.** Venn Diagram representing the CYPs and GSTs identified by a differential expression analysis and by the WGCNA modules highly correlated with the phenotypes for NTSR3 (**A**) and NTSR2 (**B**) populations. HS: sensitive population, NTSR3: derived from HS and selected for resistance to fenoxaprop-P-ethyl, NTSR2: derived from HS and selected for resistance to pendimethalin.

## Supplementary Tables captions

**Table S1**. Specific primers used for quantitative real-time PCR.

**Table S2**. Trinity assembly stats.

**Table S3.** Transdecoder assembly stats and salmon mapping rates and annotations. Rothamsted corresponding with the HS population, Fenoxaprop corresponding to the NTSR3 (derived from HS and selected for resistance to fenoxaprop-P-ethyl) and Pendimethalin corresponding to the NTSR2 (derived from HS and selected for resistance to fenoxaprop-P-ethyl).

**Table S4**. Xenome proteins with all the ORF sequences identified as differentially expressed in NTSR3 (derived from HS and selected for resistance to fenoxaprop-P-ethyl) and NTSR2 (derived from HS and selected for resistance to pendimethalin).

**Table S5.** HUB gene list in the turquoise, pink and blue modules. HS: sensitive population, NTSR3: derived from HS and selected for resistance to fenoxaprop-P-ethyl, NTSR2: derived from HS and selected for resistance to pendimethalin.

**Table S6.** Annotations in the HUB gene lists of the three modules highly correlated with the NTSR3 and NTSR2 phenotypes. Number of unigenes for each annotation are indicated in the columns. Turquoise: Positive correlation with Fenoxaprop population. Pink: Negative correlation with NTSR3 population (derived from HS and selected for resistance to fenoxaprop-P-ethyl). Blue: Positive correlation with NTSR2 population (derived from HS and selected for resistance to pendimethalin).

**Table S7**. Unigenes annotated to the enriched GO terms of the HUB genes associated to the turquoise, pink and blue modules determined by the WGCNA analysis. HUB genes showed a gene significance > 0.2 and Module membership >0.8. HS: sensitive population, NTSR3: derived from HS and selected for resistance to fenoxaprop-P-ethyl, NTSR2: derived from HS and selected for resistance to pendimethalin.

**Table S8**. Result of the KEGG enrichment of the HUB genes belonging to the turquoise, pink and blue modules determined by the WGCNA analysis. HS: sensitive population, NTSR3: derived from HS and selected for resistance to fenoxaprop-P-ethyl, NTSR2: derived from HS and selected for resistance to pendimethalin.

**Table S9**. Result of the Geneinvestigator using the signature tool in order to compare the HUB genes (and log2 of the fold change) belonging to the turquoise, pink and blue modules determined by the WGCNA analysis against wheat, barley and rice databases. HUB genes showed a gene significance > 0.2 and Module membership >0.8. Only genes with a BLASTp E-value lower than 0.01 were considered. In addition, only 400 genes can be used in the signature tool of Geneinvestigator. HS: sensitive population, NTSR3: derived from HS and selected for resistance to fenoxaprop-P-ethyl, NTSR2: derived from HS and selected for resistance to pendimethalin.
