# Supplementary figures and images for: Non-target Site Herbicide Resistance Is Conferred by Two Distinct Mechanisms in Black-Grass (Alopecurus myosuroides)
Source: Front Plant Sci. 2021 Mar 3;12:636652. doi: 10.3389/fpls.2021.636652 (PMC7966817; doi:10.3389/fpls.2021.636652)

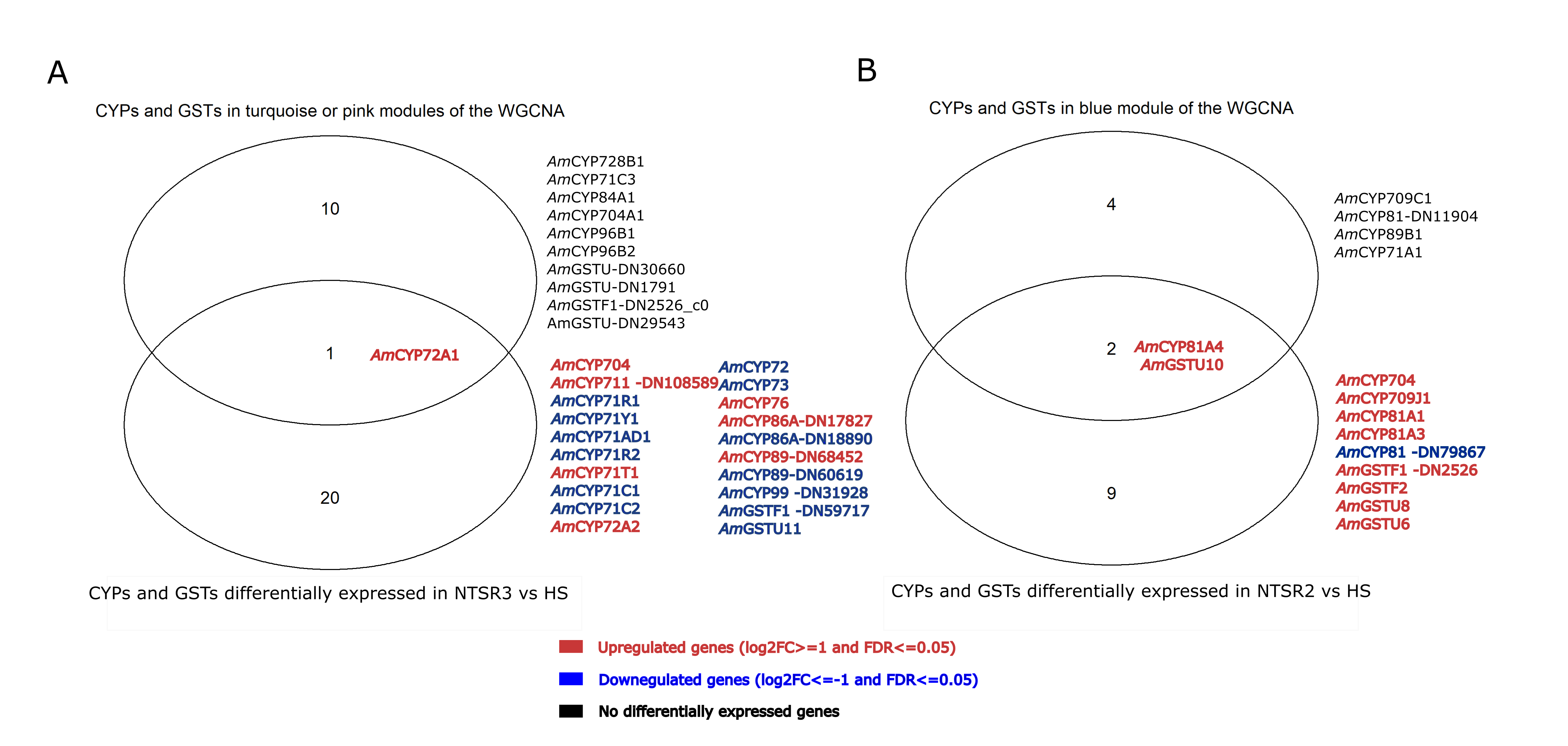

Supplement: Supplementary file 4 [file Image_2.TIFF]
